# *Echinacea purpurea* (L.) Moench: Chemical Characterization and Bioactivity of Its Extracts and Fractions

**DOI:** 10.3390/ph13060125

**Published:** 2020-06-20

**Authors:** Joana Coelho, Lillian Barros, Maria Inês Dias, Tiane C. Finimundy, Joana S. Amaral, Maria José Alves, Ricardo C. Calhelha, P. F. Santos, Isabel C.F.R. Ferreira

**Affiliations:** 1 Centro de Investigação de Montanha (CIMO), Instituto Politécnico de Bragança, Campus de Santa Apolónia, 5300-253 Bragança, Portugal; joana.coelho.16@gmail.com (J.C.); maria.ines@ipb.pt (M.I.D.); tiane@ipb.pt (T.C.F.); jamaral@ipb.pt (J.S.A.); maria.alves@ipb.pt (M.J.A.); calhelha@ipb.pt (R.C.C.); iferreira@ipb.pt (I.C.F.R.F.); 2 Centro de Química-Vila Real (CQ-VR), Universidade de Trás-os-Montes e Alto Douro, 5001-801 Vila Real, Portugal; 3 REQUIMTE/LAQV, Faculdade de Farmácia, Universidade do Porto, 4050-313 Porto, Portugal

**Keywords:** *Echinacea purpurea* (L.) Moench, phenolic compounds, polar compounds, antimicrobial activity, cytotoxicity

## Abstract

*Echinacea purpurea* (L.) Moench is widely known for its medicinal properties, being one of the most used medicinal plants for its immunostimulant properties. Nevertheless, there is still scarce information on its cytotoxic activity. Thus, this study aims at evaluating the cytotoxicity and antimicrobial activity of several aqueous and organic extracts of the aerial parts of this plant and chemically characterizing the obtained extracts. The analysis was performed by HPLC–DAD–ESI/MS. Fifteen compounds were identified; of these, seven were phenolic acids and eight were flavonoids. Non-polar compounds were evaluated by GC/MS, with a total of sixty-four compounds identified, and the most abundant groups were the sterols, fatty acids and long-chain hydrocarbons. The highest antimicrobial activity was exhibited by the dichloromethane, ethyl acetate, and acetone extracts. Dichloromethane and *n*-hexane extracts showed the highest cytotoxic activity. Therefore, they were fractionated, and the obtained fractions were also assessed for their cytotoxicity. Notwithstanding, the cytotoxicity of the extracts was superior to that of the obtained fractions, evidencing a possible synergistic effect of different compounds in the whole extracts.

## 1. Introduction

Taking advantage of the traditional ethnomedicinal application of a wide diversity of plants, they are now being used as a powerful tool for disease prevention [1]. Nowadays, approximately 30% of the pharmaceutical market and 11% of essential drugs (considered drugs intended for primary care) are plant-based [2]. *Echinacea purpurea* (L.) Moench is a perennial plant native to eastern North America that belongs to the Asteraceae family [3]. This plant species is considered a safe herbal medicine, thus, usually being used in a self-medication manner, mainly through aqueous or ethanolic extracts of the dried aerial parts or roots [4,5]. *E. purpurea* is considered one of the most known and used medicinal plants against a variety of treatments, such as snake bites and wound infections, and also for its anti-inflammatory, antioxidant and antitumor properties [6,7,8,9]. This plant can be used as an infusion or tincture, and it is available on the market in standardized preparation solutions (fluid forms) or in the form of capsules (containing dried *E. purpurea*) [10,11]. According to previous studies, the most common phytochemicals found in *E. purpurea* are alkamides, polysaccharides, lipoproteins, betaine, sesquiterpenes, polyacetylene, saponins and phenolic compounds (echinacoside and other caffeic acid derivatives, and chicoric acid) [6,11,12]. These classes of bioactive compounds have been described as being responsible for the mentioned biological properties. The multiple activities of this plant species indicate that several compounds may contribute to its medicinal benefits, which are also dependent on the plant part used, since the roots have been described as having more alkamides while the leaves are rich sources of flavonoids [13]. Nevertheless, so far, most studies on the bioactive properties of *E. purpurea* aerial parts have mainly focused on the evaluation of their immunostimulant capacity [14,15,16,17,18]. Thus, the present work aims at assessing the phenolic compounds profile of a commercial sample of *E. purpurea* aerial parts, and also at evaluating the antimicrobial and antiproliferative activity of different aqueous and organic extracts. Moreover, the most active extracts in terms of cytotoxic effects were fractionated by gradient elution through column chromatography on silica gel and the resulting fractions were assessed for their antiproliferative potential.

## 2. Results and Discussion

### 2.1. HPLC–DAD–ESI/MS Analysis of Phenolic Compounds

**Figure 1:**
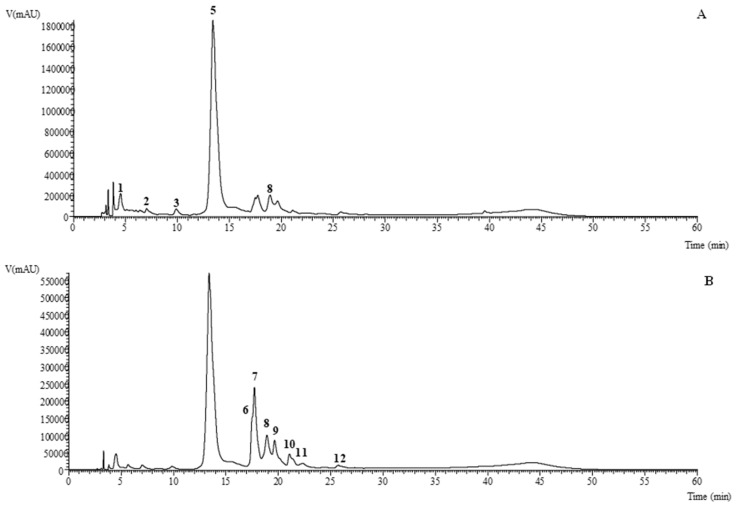
Phenolic profile of the MeOH extract of *E. purpurea* recorded at 280 nm (**A**) and 370 nm (**B**).

Table 1 presents the data (retention time, λ_max_, pseudomolecular ions, main fragment ions in MS^2^, tentative identification and quantification) obtained from the HPLC–DAD–ESI/MS analysis of the EtOAc, acetone, MeOH, infusion and decoction extracts of *E. purpurea*. An exemplificative chromatogram of the phenolic profile recorded at 280 and 370 nm of the methanolic extract is shown in Figure 1 (the extract that presented most of the phenolic compounds tentatively identified). The *n*-hexane and DCM extracts were not considered for this analysis, since in non-polar or lower polarity extracts, it is not expected to detect phenolic compounds in significant amounts. Fifteen phenolic compounds were tentatively identified, namely seven phenolic acids (mainly caffeic, chicoric, *p*-coumaric, ferulic, and caftaric acid derivatives, corresponding to peaks 1, 2, 3, 4, 5, 8 and 14) and eight flavonoids (mainly quercetin, kaempferol, and diosmetin glycosylated derivatives, corresponding to peaks 6, 7, 9, 10, 11, 12, 13 and 15). Peaks 2, 3 and 7 were identified as 5-*O*-caffeoylquinic acid, caffeic acid and quercetin-3-*O*-rutinoside, respectively, according to their retention time, mass and UV–Vis characteristics in comparison with commercial standards. Other authors also found similar compounds in different species [19]. All the remaining compounds were tentatively identified according to their mass and UV–Vis characteristics in comparison with information found in the literature. Peaks 1 ([M-H]^−^ at *m/z* 311) and 4 and 5 (both presenting [M-H]^−^ at *m/z* 473) were tentatively identified as caftaric acid and *cis* and *trans* chicoric acid, respectively, according to their pseudomolecular ion, MS^2^ fragmentation and UV–Vis spectra. *trans*-Chicoric acid was the main compound found in the methanolic extract; however, in the infusion and the decoction, the *cis* form was found in higher amounts. Peaks 8 ([M-H]^−^ at *m/z* 487) and 14 ([M-H]^−^ at *m/z* 501) were tentatively identified as feruloylcaffeoyltartaric acid and *p*-coumaroylsinapoyltartaric acid, respectively, following the identification described by Ma et al. [20]. These type of compounds have been previously reported in *E. purpurea* water:methanol:formic acid (80:20:0.1, *v/v/v*) extracts [21].

Regarding the flavonoids identified in *E. purpurea* samples, kaempferol derivatives were the main compounds found in these samples. Peaks 9, 10, 11, and 13 ([M-H]^−^ at *m/z* 607) presented a unique MS^2^ fragment at *m/z* 285, corresponding to kaempferol aglycone, which was confirmed by its characteristic UV–Vis spectrum. Peaks 9, 10 and 11 showed a pseudomolecular ion [M-H]^−^ at *m/z* 593, all being tentatively identified as kaempferol-*O*-deoxyhexosyl-hexoside, while compound 10 was identified as kaempferol-3-*O*-rutinoside, showing a similar retention time to the commercial standard. Peak 13 presented a pseudomolecular ion [M-H]^−^ at *m/z* 607 and was tentatively identified as kaempferol-*O*-deoxyhexosyl-glucuronide.

Regarding the quercetin derivatives, peak 6 presented the same chromatographic characteristics as peak 7, except for its retention time, and was tentatively identified as quercetin-*O*-deoxyhexosyl-hexoside. Peak 12 ([M-H]^−^ at *m/z* 549) revealed MS^2^ fragments at *m/z* 505 (44 u), 463 (42 u), and 301 (162 u, quercetin aglycone), and was tentatively identified as quercetin-*O*-malonylhexoside. Finally, peak 15 ([M-H]^−^ at *m/z* 577) showed an MS^2^ fragment at *m/z* 299 (diosmetin moiety). Given its higher value of retention time, the −146 u should correspond to the loss of a *p*-coumaroyl moiety and the −132 u to a pentosyl moiety, being tentatively identified as diosmetin-*O*-*p*-coumaroyl-pentoside.

Comparing the profile obtained for the different extracts, the methanolic extract was the one that presented the highest number of phenolic compounds identified, being also the extract richest in phenolic acids (53 ± 1 mg per g of extract, mainly due to *trans*-chicoric acid) and flavonoids (8.6 ± 0.2 mg per g of extract, mainly due to quercetin-3-*O*-rutinoside). The infusion and decoction showed a similar qualitative profile, with the only difference being the presence of 5-*O*-caffeoylquinic acid in the former, despite being in low amounts. Only two compounds were identified in the acetone extract, with the particularity of this extract being the only one exhibiting the presence of diosmetin-*O*-p-coumaroyl-pentoside. The EtOAc extract was found to have only minute amounts of one compound (caffeic acid), possibly due to the low polarity of the extracting solvent. According to Brown et al. [22], methanolic extracts of the *E. purpurea* are rich in chicoric acid and caftaric acid.

### 2.2. GC/MS Analysis of Non-Polar Extracts

Table 2 presents the data obtained from the GC/MS analysis of the *n*-hexane and DCM extracts, which enabled the identification of 61–63% of the compounds, corresponding to a total of 64 compounds. Among those, 35 compounds were found to be present in only one of the extracts, 17 being identified in the *n*-hexane extract and 18 in the DCM extract. In general, the extracts revealed the presence of various bioactive compounds. Regarding the terpenes group (mono, di and sesquiterpenes), they were present in the highest amounts in the *n*-hexane extract, with oxygen-containing terpenes being found only in this extract. Phytol was present in both of the *n*-hexane and DCM extracts, but in different quantities (1.1% and 0.55%, respectively). The most abundant compounds identified in the *n*-hexane extract were fatty acids (25.8% of the extract) including hexadecanoic acid (palmitic acid, C16:0), 9,12-octadecadienoic acid (linoleic acid, C18:3), octadecanoic acid (stearic acid, C18:0), followed by long-chain hydrocarbons (14.6%) and sterols (13.9%). The obtained results are in agreement with those that reported the prevalence of unsaturated fatty acids, mainly linoleic acid, in the fatty-oil of *E. purpurea* fruits obtained by extraction with *n*-hexane [23]. Regarding the DCM extract, the most abundant class of identified compounds were long-chain hydrocarbons (27.1%) and fatty acids (27.8%).

### 2.3. Biological Properties

#### 2.3.1. Antimicrobial Activity

The results concerning the ability of all seven extracts of *E. purpurea* to inhibit the growth or kill the assayed microorganisms are presented in Table 3. All tested extracts did not evidence a microbicide activity at the tested concentrations. Nevertheless, several were able to inhibit the growth of bacteria, with MICs varying between 2.5 to 20 mg/mL. The exception was the infusion extract, which did not present any activity, even at the highest tested concentration (20 mg/mL). Additionally, the decoction and the methanolic extract also evidenced low activity, with the former being able to inhibit only the growth of *K. pneumoniae* and the last only *S. aureus* (both susceptible and resistant strains). In general, the DCM, EtOAc, and acetone extracts were the ones evidencing the best antimicrobial activity, since they were capable of inhibiting the growth of all microorganisms. DCM extract showed particularly good results against Gram-positive bacteria, with *Enterococcus faecalis* and *Listeria monocytogenes* being the most susceptible ones, with a MIC value of 2.5 mg/mL. Concerning *S. aureus*, the strain MSSA was mostly inhibited by the acetone extract, with a MIC value of 2.5 mg/mL, while the strain MRSA was most sensitive to the DCM, EtOAc, and acetone extracts (all presenting a similar MIC value of 5 mg/mL). According to Hudson et al. [10], preparations of *E. purpurea* have relatively little effect on the growth of MRSA or MSSA, which was not demonstrated in the present work, since all organic extracts were able to inhibit the growth of both *S. aureus* strains, with the best results being obtained for the acetone extract. The *n*-hexane extract also evidenced the capacity of inhibiting some Gram-positive bacteria, although requiring higher MICs in general. Among the Gram-negative bacteria, the most susceptible were *Morganella morganii* and *Escherichia coli,* namely due to the DCM and EtOAc extracts, both with a MIC value of 5 mg/mL. Sharma, et al. [24] evaluated the antimicrobial activity of *E. purpurea* commercial extracts (corresponding to a mixture of roots and aerial parts extracted with 40% of EtOH) and concluded that several bacteria, including *E. coli*, *K. pneumoniae*, *P. aeruginosa* and *E. faecalis*, were relatively insensitive to the extracts, as only a minute reduction in colony forming units was obtained. In the present work, these four bacteria showed variable MIC values according to the assayed extract, with all of them having lower sensitivity (in general, MIC > 20 mg/mL) for the aqueous and methanolic extracts, while presenting better results for the less polar extracts. Regarding antifungal activity, *C. albicans* was most susceptible to the DCM and acetone extracts, showing a MIC of 5 mg/mL. This yeast also showed some susceptibility against the *n*-hexane and EtOAc extracts, with MIC values of 10 mg/mL. According to Barnes et al. [7], *n*-hexane extracts of *E. purpurea* roots show high activity against several yeast strains, including *Saccharomyces cerevisiae* and *Candida albicans*, which is in good agreement with the results obtained in the present work.

#### 2.3.2. Cytotoxic Properties of Extracts and Fractions

The results obtained regarding the cytotoxic activity of the seven extracts of *E. purpurea* are shown in Table 4. Almost all extracts showed activity against the four human tumor cell lines, with DCM being the extract that showed the best results [GI_50_ = 48 ± 4 µg/mL (NCI H460), GI_50_ = 36.7 ± 0.6 µg/mL (HepG2), GI_50_ = 51 ± 4 µg/mL (HeLa) and GI_50_ = 21 ± 2 µg/mL (MCF-7)]. Nevertheless, all the organic extracts, with the exception of MeOH, displayed also toxicity against the non-tumor hepatic cell line (PLP2), although presenting higher GI_50_ values compared to those of the tumor cell lines (Table 4). Despite the MeOH extract being only effective against the HeLa (GI_50_ = 111 ± 9) µg/mL and MCF-7 tumor cell lines (GI_50_ = 76 ± 5 µg/mL), as mentioned, it presented the advantage of not showing toxicity against the PLP2 primary cell line at the maximum concentration assayed (>400 µg/mL). The infusion was able to inhibit the growth of the MCF-7 cell line and both aqueous extracts inhibit the growth of HeLa cells, however, requiring a higher GI_50_. In addition, none presented cytotoxicity against non-tumoral PLP2 cells. This result is worth noting since the aerial parts *E. purpurea* are frequently consumed in the form of infusion when used in traditional medicine.

The obtained results correlate well with the ability to inhibit the growth of cancer cell lines, reported by Aarland et al. [6], who investigated the cytotoxic capacity of the hydroalcoholic extract prepared with the aerial parts of *E. purpurea* against MCF-7, HeLa and HCT-15 cells and showed a higher toxicity for the HeLa cell line. Tsai et al. [25] tested a hydroethanolic extract prepared with the flower of *E. purpurea*, and compared it with the commercial standard compound of chicoric acid. The results showed significant inhibition of proliferation, in a dose- and time-dependent manner, in human colon cancer cells Caco-2 and HCT-116. Chicoric acid was found to decrease the telomerase activity in HCT-116 cells, inducing apoptosis in colon cancer cells. In the present work, despite chicoric acid being also the main compound in the aqueous extracts, these extracts were the ones showing the lower cytotoxic activity. However, better results were obtained for the MeOH compared to both aqueous extracts, which can be related to its significantly higher concentration in *trans*-chicoric acid. Previously, Chicca, et al. [26] reported that one constituent of *Echinacea pallida* roots, namely the acetylenic compound pentadeca-(8Z,13Z)-dien-11-yn-2-one, revealed a concentration-dependent cytotoxicity on several human cancer cell lines, including leukemia (Jurkat and HL-60), breast carcinoma (MCF-7), and melanoma (MeWo) cells. Nevertheless, in the present work, this compound was not identified in the extracts from the aerial parts of *E. purpurea.*

According to the obtained results (Table 4), in the present work, DCM and *n*-hexane extracts were the ones that showed the most promising cytotoxic activity. Therefore, these two *E. purpurea* extracts were selected for fractionation, and the obtained fractions were further investigated for their cytotoxicity. A total of fourteen fractions from the *n*-hexane extract (FH 1–14) and fifteen fractions from the DCM (FD 1–15) were obtained, with the results of their cytotoxicity being shown in Table 4. Almost all fractions from the *n*-hexane extract showed activity against the four human tumor cell lines, nevertheless showing a marked reduction in activity when compared to the whole extract, as the GI_50_ values were much higher. The same was observed for the DCM fractions, with the reduction in activity being more evident in this case since, besides higher GI_50_ values, five fractions did not present any cytotoxic activity at all. In both cases, the obtained results suggest a high synergic effect among the compounds present in the whole extract. Therefore, considering that all DCM and *n*-hexane fractions exhibited a lower cytotoxic activity compared to the whole extract, they were not further analyzed for its chemical composition. In what concerns the two whole extracts, DCM and *n*-hexane, by comparing their chemical composition obtained by GC–MS analysis (Table 2), it can be observed that the composition regarding the major group of compounds is somehow similar, comprising fatty acids, sterols, and long-chain hydrocarbons, thus, not justifying the differences observed in the cytotoxic assays. However, it should be noticed that, for both extracts, several compounds were not possible to be identified by the used technique (corresponding to 37.2% and 38.9% for DCM and *n*-hexane extracts, respectively). Most possibly, those non-identified compounds can be related with both the cytotoxic effects exhibited against the tumor and hepatic cell lines as well as the synergic effects suggested by the results obtained with the fractions.

Todd et al. [16] evaluated the effect of a 75% ethanolic extract of *E. purpurea* roots, and thereof, fractions on the suppression of cytokines and observed that both the fractions that contained alkylamides as well as those without detectable alkylamides displayed similar suppressive effects, allowing the authors to conclude that the ethanolic *E. purpurea* extract probably contains multiple constituents that differentially regulate cytokine production by macrophages.

## 3. Materials and Methods

### 3.1. Standards and Reagents

Acetonitrile 99.9% was of HPLC grade was obtained from Fisher Scientific (Lisbon, Portugal). Phenolic compound standards were obtained from Extrasynthese (Genay, France). Formic acid was obtained from Sigma-Aldrich (St. Louis, MO, USA). Water was treated in a Milli-Q water purification system (TGI Pure Water Systems, Greenville, SC, USA). Fetal Bovine Serum (FBS), solutions of penicillin (100 IU/mL) and streptomycin (10 mg/mL), RPMI-1640 medium, trypsin-EDTA (ethylenediaminetetraacetic acid), L-glutamine and Hank’s Balanced Salt Solution (HBSS) were purchased from Hyclone (Logan, USA). Acetic acid, sulforhodamine B (SRB), trypan blue, trichloroacetic acid (TCA) and Tris-base were purchased from Sigma Chemical Co. (Saint Louis, USA). Silica gel 0.060–0.200 mm 60 A was obtained from Acros Organics (Geel, Belgium).

### 3.2. Plant Material

*Echinacea purpurea* (L.) Moench (aerial parts) (150 g) were acquired from Cantinho das Aromáticas, Vila Nova de Gaia, Portugal, in September 2017. According to the supplier, *E. purpurea* was from a biological production, harvested when flowering and dried at 40–45 °C for three days in a dryer with controlled ventilation. The dried sample was ground to a fine powder, mixed to obtain a homogeneous sample and stored at room temperature protected from light and humidity.

### 3.3. Preparation of Extracts

The extracts were prepared from the powdered plant using different solvents with increasing polarities, as described by Graça et al. [27]. The extracts were separated into organic (*n*-hexane, dichloromethane—DCM, ethyl acetate—EtOAc, acetone, and methanol—MeOH) and aqueous (decoction and infusion) extracts.

#### 3.3.1. Organic Extracts

For the preparation of the mentioned organic extracts, 7 g of dried plant material were submitted to a sequential extraction process based on increasing the solvent’s polarity. The plant was extracted twice with each organic solvent (500 mL) for 48 h, under vigorous stirring (150 rpm), at room temperature. The solutions were filtered under reduced pressure through a sintered Buchner glass funnel, the combined organic extracts were evaporated to dryness under reduced pressure at 40 °C (Büchi R-20, Flawil, Switzerland), and the obtained residue was further extracted with another solvent following the same procedure.

#### 3.3.2. Aqueous Extracts

Two aqueous extracts were prepared: decoction and infusion. For the former, the dried sample (1 g) was added to 100 mL of distilled water and boiled for 5 min; the infusion extract was obtained by adding the dried sample (1 g) in 100 mL of boiling water and left to stand for 5 min at room temperature. The mixtures were filtered under reduced pressure through a sintered glass Buchner funnel, and further frozen and lyophilized.

### 3.4. Fractionation of the Extracts

Fractionation was carried out in the extracts that showed promising results in the bioactivity assays, namely DCM and *n*-hexane extracts. The procedure was conducted based on the methodology described by Graça et al. [28], with some modifications.

#### 3.4.1. Dichloromethane Extracts

The extract was diluted in the minimum amount of CH_2_Cl_2_, and a small amount of silica gel was added. The mixture was evaporated to dryness at 40 °C under reduced pressure and, afterwards, placed on the top of a silica gel column. The dry-loaded extract was fractionated by gradient elution column chromatography (20 × 400 mm) using: CH_2_Cl_2_; CH_2_Cl_2_/EtOAc—(9:1), (8:2), (7:3), (6:4), (5:5), (4:6), (3:7), (2:8), (1:9); EtOAc; EtOAc/acetone—(9:1), (8:2), (7:3), (6:4), (5:5), (6:4), (7:3), (8:2), (9:1); acetone; acetone/MeOH—(9:1), (8:2), (7:3), (6:4), (5:5), (4:6), (3:7), (2:8), (1:9); MeOH; MeOH/formic acid (99:1), (97:3), (95:5). A total of seven hundred and twenty-four eluates (∼23 mL each) were collected and grouped into fifteen fractions (FD 1–15), according to the similarity of their TLC profiles [silica gel, CH_2_Cl_2_/MeOH—(99:1), stained with 50% H_2_SO_4_ in MeOH, heating]. The solvent of these final fractions was removed under reduced pressure until dryness.

#### 3.4.2. *n*-Hexane Extracts

For the *n*-hexane extract, a fractionation procedure similar as described for the DCM extract was used. The gradient elution using the same solvents/mixture of solvents allowed collecting of a total of eight hundred and thirty-six samples (~23 mL each), which were collected and grouped in fourteen fractions (FH 1–14), according to the similarity of their TLC profiles [silica gel, CH_2_Cl_2_/MeOH—(99:1), stained with 50% H_2_SO_4_ in MeOH, heating]. The solvent of these final fractions was removed under reduced pressure until complete dryness.

### 3.5. Chemical Characterization

#### 3.5.1. Analysis of Phenolic Compounds by HPLC–DAD–ESI/MS

The phenolic compounds present in *E. purpurea* extracts were analyzed as described in Bessada et al. [29], with minor modifications. The EtOAc and acetone extracts were dissolved in MeOH, while the methanol extract was dissolved in MeOH/H_2_O (1:4, *v/v*), and the infusion and the decoction extracts were dissolved in distilled H_2_O, at a final concentration of 5 mg/mL, and filtered through a 0.22 µm disposable LC filter disk. All the extracts were analyzed using a Dionex Ultimate 3000 UPLC (Thermo Scientific, San Jose, CA, USA) system equipped with a diode array detector coupled to an electrospray ionization mass detector (LC-DAD-ESI/MS^n^), a quaternary pump, an autosampler (kept at 5 °C), a degasser and an automated thermostatized column compartment. Chromatographic separation was achieved with a Waters Spherisorb S3 ODS-2 C18 (3 μm, 4.6 × 150 mm, Waters, Milford, MA, USA), column thermostatized at 35 °C. The solvents used were: (A) 0.1% formic acid in water, (B) acetonitrile. The elution gradient established was the following: 15% B (5 min), 15% B to 20% B (5 min), 20–25% B (10 min), 25–35% B (10 min), 35–50% B (10 min), and re-equilibration of the column, using a flow rate of 0.5 mL/min. Double online detection was carried out in the DAD (using 280, 330 and 370 nm as preferred wavelengths) and in a mass spectrometer (MS) connected to a HPLC system via the DAD cell outlet. MS detection was performed in the negative mode, using a Linear Ion Trap LTQ XL mass spectrometer (Thermo Finnigan, San Jose, CA, USA) equipped with an ESI source. Nitrogen served as the sheath gas (50 psi); the system was operated with a spray voltage of 5 kV, a source temperature of 325 °C, and a capillary voltage of −20 V. The tube lens offset was kept at a voltage of −66 V. The full scan covered the mass range from *m/z* 100 to 1500. The collision energy used was 35 (arbitrary units). Data acquisition was carried out with Xcalibur^®^ data system (Thermo Finnigan, San Jose, CA, USA). The phenolic compounds were identified by comparing their retention times, UV–Vis and mass spectra with those obtained from standard compounds, when available. Otherwise, compounds were tentatively identified comparing the obtained information with available data reported in the literature. For quantitative analysis, 7-level calibration curves for each available phenolic standard was constructed based on the UV signal of caffeic acid (*y* = 388345*x* + 406369, *R^2^* = 0.999, LOD = 0.78 µg/mL and LOQ = 1.97 µg/mL), ferulic acid (*y* = 633126*x* − 185462, *R^2^* = 0.999, LOD = 1.85 µg/mL and LOQ = 5.61 µg/mL), *p*-coumaric acid (*y* = 301950*x* + 6966.7, *R^2^* = 0.9999, LOD = 1.10 µg/mL and LOQ = 3.32 µg/mL), and quercetin-3-*O*-rutinoside (*y* = 13343*x* + 76751, *R^2^* = 0.999, LOD = 0.21 µg/mL and LOQ = 0.71 µg/mL). For the identified phenolic compounds, for which a commercial standard was not available, the quantification was performed through the calibration curve of the most similar available standard (Figure S1, Supplementary Material). The results were expressed as mg per g of extract.

#### 3.5.2. Analysis of Non-Polar Compounds by GC–MS

*E. purpurea n*-hexane and DCM extracts were chemically characterized by GC–MS after sample derivatization. A portion of the *N*-hexane and DCM extracts (50 mg) was dissolved in 600 μL of bis-(trimethylsilyl) trifluoroacetamide (BSTFA; PanReac AppliChem, Germany) and the mixture was heated at 70 °C for 1 h. The derivatized sample was analyzed by GC–MS using a GC-2010 Plus (Shimadzu, USA) gas chromatography system equipped with a AOC-20iPlus (Shimadzu) automatic injector, a SH-RXi-5ms column (30 m × 0.25 mm × 0.25 μm; Shimadzu, USA) and a mass spectrometry detector, operating under the conditions previously described by Spréa et al. [30], with minor modifications, namely the oven program, was as follows: initial oven temperature of 45 °C increasing at 3 °C/min to 175 °C, then at 15 °C/min to 300 °C, and finally, was held isothermal for 15 min. The compounds were identified based on the comparison of the obtained spectra with those from the NIST17 mass spectral library, and confirmed by determining the linear retention index (LRI) based on the retention times of an *N*-alkanes mixture (C8–C40, Supelco). Whenever possible, comparisons were also performed with commercial standards and with published data. Compounds were quantified as relative percentage using relative peak area values obtained directly from the total ion current (TIC) values.

### 3.6. Evaluation of the Bioactive Properties

#### 3.6.1. Antimicrobial Activity

The organic and aqueous extracts were evaluated for their antimicrobial potential based on the methodology presented by Alves, et al. [31], with some modifications. The used microorganisms were clinical isolates from patients hospitalized in various departments of the Centro Hospitalar de Trás-os-Montes e Alto Douro (Vila Real and Bragança) and comprised five Gram-negative bacteria (*Escherichia coli, Klebsiella pneumoniae, Morganella morganii, Proteus mirabilis and Pseudomonas aeruginosa*), three Gram-positive bacteria (*Enterococcus faecalis, Listeria monocytogenes*, Methicillin-resistant *Staphylococcus aureus* (MRSA), and Methicillin-Susceptible *Staphylococcus aureus* (MSSA)) and a yeast (*Candida albicans*). All the microorganisms were incubated at 37 °C in an appropriate fresh medium for 24 h before analysis to maintain the exponential growth phase. The minimum inhibitory concentration (MIC), corresponding to the lowest concentration of the *E. purpurea* extracts able to inhibit microbial growth, was determined against each microorganism using a colorimetric assay. The extracts were dissolved in 5% (*v/v*) dimethyl sulfoxide (DMSO)/Mueller–Hinton Broth (MHB) or Tryptic Soy Broth (TSB) to give a final concentration of 100 mg/mL for the stock solution. Then, the samples were serially diluted, obtaining a concentration range from 20 to 0.15 mg/mL. For the determination of the minimal bactericide concentration (MBC) or minimal fungicidal concentration (MFC) for the yeast, 10 μL of each well that showed no change in color was plated on blood agar (7% sheep blood) solid medium and incubated at 37 °C for 24 h. The lowest concentration that yielded no growth was set as the MBC or MFC. A negative control was prepared with 5% (*v/v*) dimethyl sulfoxide (DMSO)/Mueller–Hinton Broth (MHB) or Tryptic Soy Broth (TSB). One growth control was prepared with MHB and each inoculum. For the Gram-negative bacteria, antibiotics, such as ampicillin and Imipenem, were used as positive controls, while for the Gram-positive bacteria, ampicillin and vancomycin were selected.

#### 3.6.2. Cytotoxic Activity

The evaluation of cytotoxicity was in human tumor cell lines, namely: NCI-H460 (lung cancer); MCF-7 (breast adenocarcinoma); HepG2 (hepatocellular carcinoma); HeLa (cervical carcinoma) was determined according to the procedure used in Barros et al. [32]. A phase-contrast microscope was used to monitor the growth of cell cultures, which were subcultured and plated in 96-well plates (density of 5.0 × 10^4^ cells/mL) using Dulbecco’s modified Eagle’s medium (DMEM) supplemented with FBS (10%), 1% penicillin/streptomycin. The cell growth inhibition was measured using the sulforhodamine B (SRB) assay, where the quantity of pigmented cells is directly proportional to the total protein content and, therefore, to the number of bounded cells. The samples were dissolved in water or DMSO (1%) depending on the extract, at 8 mg/mL and then, submitted to further dilutions (400–3.125 µg/mL). The results were expressed as GI_50_ values (sample concentration that inhibited 50% of the net cell growth, in μg/mL). Ellipticine was used as the positive control.

#### 3.6.3. Hepatotoxicity

For the hepatotoxicity assay, a cell culture was prepared from a freshly harvested porcine liver (obtained from a local slaughterhouse) and designated as PLP2 [33]. Before reaching confluence, cells were subcultured and plated in 96-well plates at a density of 1.0 × 10^4^ cells/well and cultivated in commercial DMEM medium supplemented with 10% FBS, 1% penicillin/streptomycin. The samples were dissolved in water or DMSO (1%) depending on the extract, at 8 mg/mL, and then, submitted to further dilutions (400-3.125 μg/mL). Ellipticine was used as a positive control and results were expressed in GI_50_ values corresponding to the sample concentration achieving 50% of growth inhibition in liver primary culture PLP2.

### 3.7. Statistical Analysis

The described experiments were performed in triplicate and the results were expressed as the mean ± standard deviation (SD). The differences between the different extracts were analyzed using one-way analysis of variance (ANOVA) followed by Tukey’s honest significant difference post hoc test with α = 0.05, coupled with Welch’s statistic. A Student’s *t*-test was used to determine the significant difference among two different samples, with α = 0.05. Statistical analysis was carried out using the SPSS v. 23.0 program (SPSS v. 23.0; IBM Corp., Armonk, NY, USA).

## 4. Conclusions

The work reported herein highlights the difference of the biological activity in the extracts of *E. purpurea* prepared with solvents of different polarity as well as their chemical characterization. The highest antimicrobial activity was observed for the DCM, EtOAc, and acetone extracts, while the highest cytotoxicity was evidenced by the DCM and *n*-hexane extracts. Despite exhibiting lower activity, it is worth noting that the infusion, which is frequently used by consumers, was able to inhibit the growth of MCF-7 and HeLa cell lines. In general, the cytotoxicity of the DCM and *n*-hexane extracts was superior compared to the corresponding fractions, which points to a possible synergistic effect of the mixture of compounds present in the initial extracts.

## Figures and Tables

**Table 1:** Retention time (Rt), wavelengths of maximum absorption in the visible region (λ_max_), mass spectral data, tentative identification and quantification (mg/g extract) of the phenolic compounds present in five different extracts of *E. purpurea*.

Peak	Rt (min)	λ_max_ (nm)	[M-H]^−^ (*m/z*)	MS^2^ (*m/z*)	Tentative Identification	Organic			Aqueous	
						EtOAc	Acetone	MeOH	Infusion	Decoction
1	4.54	327	311	179(6),149(100),135(5),113(3)	Caftaric acid	nd	nd	1.19 ± 0.02 ^a^	0.16 ± 0.01 ^c^	0.22 ± 0.01 ^b^
2	7.04	325	353	191(100),179(8),173(3),135(3)	5-*O*-caffeoylquinic acid	nd	nd	0.286 ± 0.003 ^a^	0.024 ± 0.001 ^b^	nd
3	9.87	324	179	135(100)	Caffeic acid	0.20 ± 0.01 ^b^	0.066 ± 0.001 ^c^	0.63 ± 0.02 ^a^	nd	nd
4	11.84	328	473	311(100),293(87),179(5),149(5)	*cis* Chicoric acid	nd	nd	nd	12.0 ± 0.1 *	10.5 ± 0.1 *
5	13.40	328	473	311(100),293(87),179(5),149(5)	*trans* Chicoric acid	nd	nd	41.0 ± 0.3 ^a^	4.0 ± 0.1 ^b^	4.5 ± 0.1 ^b^
6	17.50	342	609	301(100)	Quercetin-*O*-deoxyhexosyl-hexoside	nd	tr	1.8836 ± 0.0005	nd	nd
7	17.73	342	609	301(100)	Quercetin-3-*O*-rutinoside	nd	tr	5.6 ± 0.2 ^a^	1.4 ± 0.1 ^b^	0.26 ± 0.01 ^c^
8	18.93	328	487	325(85),307(51),293(100),193(10),179(15)	Feruloylcaffeoyltartaric acid	nd	0.030 ± 0.001 ^d^	1.36 ± 0.04 ^a^	0.10 ± 0.01 ^b^	0.040 ± 0.001 ^c^
9	19.66	329	593	285(100)	Kaempferol-*O*-deoxyhexosyl-hexoside	nd	tr	0.60 ± 0.01	nd	tr
10	21.10	334	593	285(100)	Kaempferol-3-*O*-rutinoside	nd	tr	0.536 ± 0.001	nd	tr
11	21.42	334	593	285(100)	Kaempferol-*O*-deoxyhexosyl-hexoside	nd	tr	0.058 ± 0.003	nd	nd
12	22.35	327	549	505(5),463(13),301(30)	Quercetin-*O*-malonylhexoside	nd	nd	nd	nd	nd
13	24.10	340	607	285(100)	Kaempferol-*O*-deoxyhexosyl-glucuronide	nd	tr	nd	nd	nd
14	25.73	327	501	337(100),307(50),277(15),233(61),203(5)	*p*-Coumaroylsinapoyltartaric acid	nd	nd	0.050 ± 0.001 ^a^	0.010 ± 0.001 ^b^	0.010 ± 0.001 ^b^
15	39.56	308	577	299(100)	Diosmetin-*O*-*p*-coumaroyl-pentoside	nd	5.29 ± 0.04	nd	nd	nd
					Total phenolic acids	0.20 ± 0.01 ^b^	0.066 ± 0.001 ^a^	44.5 ± 0.4 ^b^	16.4 ± 0.2 ^b^	15.2 ± 0.2 ^b^
					Total flavonoids	nd	5.29 ± 0.04 ^b^	8.6 ± 0.2 ^a^	1.4 ± 0.1 ^c^	0.26 ± 0.01 ^d^
					Total phenolic compounds	0.20 ± 0.01 ^e^	5.36 ± 0.04 ^d^	53 ± 1 ^a^	17.8 ± 0.1 ^b^	15.5 ± 0.2 ^c^

Nd—not detected. tr—traces. * Samples differ significantly (*p* < 0.05), obtained by Student’s *t*-test. Results expressed in mean values ± standard deviation (SD). Different letters represent significant differences (*p* < 0.05).

**Table 2:** Chemical composition of *n*-hexane and DCM extracts of *E. purpurea* obtained by GC–MS analysis.

Number	Compound	RT (min)	LRI ^a^	Relative % ^b^		*t*-Students Test *p*-Value
				*n*-Hexane	DCM	
1	2,3-Butanediol	14.97	1044 *	-	0.055 ± 0.003	-
2	Hexanoic acid	16.56	1079 *	0.017 ± 0.004	0.061 ± 0.001	<0.001
3	2-Methyl-4-pentenoic acid	16.83	1085 *	-	0.027 ± 0.002	-
4	(*E*)-2-Hexenoic acid	18.83	1127 *	-	0.018 ± 0.002	-
5	Verbenone	22.92	1215	0.009 ± 0.001	-	-
6	(+)-*cis*-Verbenol	24.11	1241 *	0.009 ± 0.002	-	-
7	Benzoic acid	24.65	1253 *	-	0.039 ± 0.003	-
8	Menthol	25.24	1264 *	0.0035 ± 0.0004	-	-
9	Octanoic acid	25.52	1270 *	0.013 ± 0.003	0.026 ± 0.002	<0.001
10	Carvacrol	28.54	1340 *	0.13 ± 0.04	0.1256 ± 0.00004	0.155
11	Copaene	30.49	1385	0.013 ± 0.001	-	-
12	β-Caryophyllene	32.39	1430	0.034 ± 0.002	-	-
13	Decanoic acid	33.91	1466 *	0.198 ± 0.001	0.125 ± 0.002	<0.001
14	D-(-)-Citramalic acid	35.04	1494 *	-	0.077 ± 0.003	-
15	Epicubebol	35.47	1504	0.048 ± 0.003	-	-
16	Malic acid	35.79	1512 *	0.033 ± 0.002	0.115 ± 0.002	<0.001
17	Dihydroactinidiolide	36.83	1539	0.052 ± 0.002	-	-
18	*trans*-Nerolidol	38.09	1571	0.048 ± 0.001	-	-
19	Spathulenol	38.79	1589	0.22 ± 0.01	-	-
20	Caryophyllene oxide	39.03	1595	0.22 ± 0.01	-	-
21	Dodecanoic acid	41.55	1662 *	0.15 ± 0.04	0.066 ± 0.002	<0.001
22	Oplopanone	44.49	1755	0.507 ± 0.004	-	-
23	Azelaic acid	45.78	1803 *	-	0.371 ± 0.003	-
24	Neophytadiene	46.35	1845	0.907 ± 0.001	2.71 ± 0.1	<0.001
25	Myristic acid	46.53	1858 *	1.4 ± 0.1	1.14 ± 0.01	0.092
26	Hexadecanoic acid, methyl ester	47.51	1936	0.351 ± 0.004	-	-
27	Pentadecanoic acid	47.78	1960 *	0.44 ± 0.02	0.27 ± 0.01	<0.001
28	Gallic acid	48.09	1988 *	-	0.12 ± 0.01	-
29	Undecanedioic acid	48.29	2006 *	-	0.154 ± 0.003	-
30	Palmitelaidic acid	48.58	2039 *	0.16 ± 0.01	-	-
31	Palmitic Acid	48.79	2061 *	4.6 ± 0.1	5.7 ± 0.2	0.015
32	Linoleic acid, methyl ester	49.21	2109	0.644 ± 0.001	-	-
33	Heptadecanoic acid	49.59	2157 *	0.8 ± 0.1	-	-
34	Caffeic acid	49.62	2161 *	-	0.822 ± 0.003	-
35	Phytol	49.85	2190 *	1.1 ± 0.1	0.55 ± 0.01	<0.001
36	Docosane	49.99	2210	-	0.42 ± 0.02	-
37	Linoleic acid	50.12	2229 *	5.0 ± 0.1	4.3 ± 0.1	0.016
38	α-Linolenic acid	50.17	2236 *	2.2 ± 0.2	2.4 ± 0.1	0.121
39	Stearic acid	50.31	2256 *	2.30 ± 0.04	1.5 ± 0.1	<0.001
40	Tricosane	50.68	2311	-	1.0 ± 0.1	-
41	Tetracosane	51.30	2411	1.6 ± 0.1	1.9 ± 0.1	0.025
42	Arachidic acid	51.55	2454 *	3.3 ± 0.2	1.664 ± 0.005	<0.001
43	Pentacosane	51.89	2511	1.6 ± 0.02	3.20 ± 0.04	<0.001
44	Heneicosanoic acid	52.13	2552 *	0.37 ± 0.02	0.56 ± 0.04	<0.001
45	Hexacosane	52.48	2612	1.58 ± 0.01	4.36 ± 0.02	<0.001
46	Behenic acid	52.73	2651 *	1.21 ± 0.05	1.4 ± 0.1	0.084
47	3-Methylhexacosane	52.95	2686	-	0.47 ± 0.02	-
48	Heptacosane	53.12	2712	2.6 ± 0.1	5.0 ± 0.3	<0.001
49	Tricosanoic acid	53.39	2750 *	0.82 ± 0.03	0.84 ± 0.02	0.373
50	Octacosane	53.83	2812	1.6 ± 0.01	4.4 ± 0.1	<0.001
51	Lignoceric acid	54.12	2849 *	1.3 ± 0.1	1.04 ± 0.01	0.023
52	Squalene	54.18	2856	1.3 ± 0.1	0.434 ± 0.02	<0.001
53	2-Methyl-octacosane	54.31	2873	-	0.592 ± 0.01	-
54	3-Methyloctacosane	54.41	2886	-	0.52 ± 0.01	-
55	Nonacosane	54.63	2912	3.1 ± 0.1	5.14 ± 0.03	<0.001
56	1-Hexacosanol	55.01	2953 *	1.6 ± 0.1	1.3 ± 0.1	0.091
57	Hexacosanoic acid	55.95	3048 *	-	1.4 ± 0.1	-
58	Nonacosanal	56.07	3059 *	0.75 ± 0.04	-	-
59	Untriacontane	56.67	3112	2.4 ± 0.2	-	-
60	α-Tocopherol	57.56	3182	-	1.09 ± 0.04	-
61	Cholesterol	58.11	3221 *	0.39 ± 0.01	-	-
62	Campesterol	59.78	3325 *	2.00 ± 0.01	0.41 ± 0.02	<0.001
63	Stigmasterol	60.31	3353 *	3.9 ± 0.2	-	-
64	β-Sitosterol	61.40	3416 *	7.5 ± 0.5	4.8 ± 0.3	<0.001
	Total identified compounds			61.0 ± 0.5	63 ± 1	0.482
	Monoterpene hydrocarbons			0.06 ± 0.002	-	-
	Oxygen-containing monoterpenes			0.021 ± 0.002	-	-
	Sesquiterpene hydrocarbons			0.38 ± 0.03	0.1256 ± 0.00004	<0.001
	Oxygen-containing sesquiterpenes			0.824 ± 0.002	-	-
	Diterpene hydrocarbons			3.4 ± 0.2	3.7 ± 0.1	0.151
	Sterol			13.9 ± 0.3	5.2 ± 0.3	<0.001
	Fatty acids			24.8 ± 0.5	23 ± 1	0.362
	Long-chain hydrocarbons			14.57 ± 0.03	27.1 ± 0.3	<0.001
	Long-chain alcohols			1.6 ± 0.1	1.3 ± 0.1	0.018
	Others			1.4 ± 0.04	2.61 ± 0.04	<0.001

^a^ LRI, linear retention index determined on a SH-RXi-5ms fused silica column relative to a series of *n*-alkanes (C8–C40). * identified as TMS derivative. ^b^ relative % is given as mean ± SD, *n* = 3. In each row, *p* values < 0.05 means significant differences.

**Table 3:** Antimicrobial activity of all seven *E. purpurea* extracts against clinical isolates of Gram-negative and Gram-positive bacteria and one yeast strain.

	Organic Extracts										Aqueous Extracts				Controls							
	*n*-Hexane		DCM		EtOAc		Acetone		MeOH		Infusion		Decoction		Ampicilin		Imipenem		Vancomycin		Fluconazol	
	MIC	MBC	MIC	MBC	MIC	MBC	MIC	MBC	MIC	MBC	MIC	MBC	MIC	MBC	MIC	MBC	MIC	MBC	MIC	MBC	MIC	MBC
Gram-negative bacteria																						
*Escherichia coli*	20	>20	10	>20	5	>20	10	>20	>20	>20	>20	>20	>20	>20	<0.15	<0.15	<0.0078	<0.0078	nt	nt	nt	nt
*Klebsiella pneumoniae*	20	>20	20	>20	10	>20	10	>20	>20	>20	>20	>20	20	>20	10	20	<0.0078	<0.0078	nt	nt	nt	nt
*Morganella morganii*	20	>20	5	>20	5	>20	10	>20	>20	>20	>20	>20	>20	>20	20	>20	<0.0078	<0.0078	nt	nt	nt	nt
*Proteus mirabilis*	20	>20	20	>20	10	>20	10	>20	>20	>20	>20	>20	>20	>20	<0.15	<0.15	<0.0078	<0.0078	nt	nt	nt	nt
*Pseudomonas aeruginosa*	20	>20	10	>20	10	>20	10	>20	>20	>20	>20	>20	>20	>20	>20	>20	0.5	1	nt	nt	nt	nt
Gram-positive bacteria																						
*Enterococcus faecalis*	10	>20	2.5	>20	10	>20	5	>20	>20	>20	>20	>20	>20	>20	<0.15	<0.15	nt	nt	<0.0078	<0.0078	nt	nt
*Listeria monocytogenes*	>20	>20	2.5	>20	20	>20	20	>20	>20	>20	>20	>20	>20	>20	<0.15	<0.15	<0.0078	<0.0078	nt	nt	nt	nt
MRSA	10	>20	5	>20	5	>20	5	>20	20	>20	>20	>20	>20	>20	<0.15	<0.15	nt	nt	<0.0078	<0.0078	nt	nt
MSSA	10	>20	5	>20	5	>20	2.5	>20	20	>20	>20	>20	>20	>20	<0.15	<0.15	nt	nt	0.25	0.5	nt	nt
Yeasts	MIC	MFC	MIC	MFC	MIC	MFC	MIC	MFC	MIC	MFC	MIC	MFC	MIC	MFC	MIC	MFC	MIC	MFC	MIC	MFC	MIC	MFC
*Candida albicans*	10	>20	5	>20	10	>20	5	>20	>20	>20	>20	>20	>20	>20	nt	nt	nt	nt	nt	nt	0.06	0.06

MIC and MBC are expressed in mg/mL. nt—not tested. The highest tested concentration was 20 mg/mL.

**Table 4:** Cytotoxicity of organic and aqueous extracts and obtained fractions (*n*-hexane and DCM, μg/mL) of *E. purpurea* against human tumor cell lines and non-tumor cell line (PLP2).

		NCI H460	HepG2	HeLa	MCF-7	PLP2
Organic	*n*-Hexane	70 ± 2 ^c^	47 ± 3 ^c^	58 ± 5 ^e^	29 ± 2 ^d^	104 ± 7 ^c^
	DCM	48 ± 4 ^d^	36.7 ± 0.6 ^c^	51 ± 4 ^e^	21 ± 2 ^e^	100 ± 8 ^c^
	EtOAc	192 ± 4 ^a^	226 ± 15 ^a^	85 ± 6 ^d^	51 ± 5 ^c^	166 ± 9 ^b^
	Acetone	142 ± 10 ^b^	82 ± 4 ^b^	98 ± 5 ^cd^	50 ± 4 ^c^	195 ± 15 ^a^
	MeOH	> 400	> 400	111 ± 9 ^c^	76 ± 5 ^b^	>400
Aqueous	Infusion	>400	>400	305 ± 23 ^b^	247 ± 5 ^a^	>400
	Decoction	>400	>400	319 ± 12 ^a^	>400	>400
Fraction of *n*-hexane	FH1	105 ± 7 ^h^	206 ± 12 ^e^	150 ± 5 ^d^	110 ± 3 ^fg^	326 ± 17 ^b^
	FH2	142 ± 5 ^g^	344 ± 10 ^ab^	180 ± 7 ^c^	140 ± 7 ^e^	>400
	FH3	184 ± 15 ^ef^	308 ± 19 ^c^	212 ± 6 ^b^	182 ± 9 ^c^	>400
	FH4	243 ± 16 ^a^	233 ± 3 ^d^	240 ± 3 ^a^	222 ± 5 ^b^	>400
	FH5	238 ± 4 ^ab^	359 ± 7 ^a^	232 ± 5 ^a^	239 ± 4 ^a^	>400
	FH6	250 ± 1 ^a^	327 ± 16 ^bc^	235 ± 5 ^a^	237 ± 8 ^a^	>400
	FH7	197 ± 12 ^de^	159 ± 6 ^gh^	213 ± 8 ^b^	179 ± 9 ^c^	343 ± 22 ^ab^
	FH8	222 ± 5 ^bc^	168 ± 5 ^fg^	241 ± 2 ^a^	121 ± 10 ^f^	356 ± 11 ^a^
	FH9	206 ± 11 ^cd^	159 ± 3 ^gh^	181 ± 8 ^c^	171 ± 4 ^cd^	342 ± 10 ^ab^
	FH10	211 ± 5 ^cd^	141 ± 4 ^hi^	170 ± 9 ^c^	181 ± 5 ^c^	331 ± 6 ^ab^
	FH11	100 ± 7 ^h^	93 ± 4 ^j^	105 ± 9 ^f^	106 ± 10 ^g^	269 ± 6 ^c^
	FH12	128 ± 5 ^g^	131 ± 6 ^i^	127 ± 9 ^e^	111 ± 3 ^fg^	>400
	FH13	168 ± 12 ^f^	187 ± 12 ^ef^	176 ± 12 ^c^	160 ± 7 ^d^	>400
	FH14	>400	338 ± 13 ^b^	>400	>400	>400
Fraction of DCM	FD1	>400	>400	>400	>400	>400
	FD2	236 ± 11 ^c^	278 ± 9 ^ab^	178 ± 12 ^c^	146 ± 11 ^c^	>400
	FD3	>400	>400	>400	>400	>400
	FD4	>400	>400	>400	>400	>400
	FD5	128 ± 4 ^e^	146 ± 4 ^f^	116 ± 4 ^e^	89 ± 8 ^d^	267 ± 13 *
	FD6	135 ± 4 ^e^	141 ± 3 ^f^	113 ± 4 ^e^	81 ± 3 ^d^	>400
	FD7	170 ± 2 ^d^	187 ± 16 ^e^	150 ± 3 ^d^	139 ± 5 ^c^	302 ± 18 *
	FD8	225 ± 9 ^c^	212 ± 3 ^d^	174 ± 9 ^c^	151 ± 4 ^bc^	>400
	FD9	238 ± 4 ^c^	205 ± 7 ^de^	178 ± 9 ^c^	144 ± 9 ^c^	>400
	FD10	341 ± 9 ^a^	298 ± 19 ^a^	241 ± 5 ^a^	181 ± 9 ^a^	>400
	FD11	291 ± 9 ^b^	253 ± 8 ^c^	220 ± 5 ^b^	149 ± 3 ^bc^	>400
	FD12	292 ± 17 ^b^	256 ± 11 ^c^	223 ± 14 ^b^	144 ± 3 ^c^	>400
	FD13	336 ± 10 ^a^	271 ± 4 ^bc^	254 ± 6 ^a^	161 ± 4 ^b^	>400
	FD14	>400	>400	>400	>400	>400
	FD15	>400	>400	>400	>400	>400

Results expressed in mean values ± standard deviation (SD). Different letters correspond to significant differences (*p* < 0.05). * Samples differ significantly (*p* < 0.05), obtained by Student’s *t*-test. Ellipticine GI_50_ values: 1.21 μg/mL (MCF-7), 1.03 μg/mL (NCI-H460), 0.91 μg/mL (HeLa), 1.10 μg/mL (HepG2) and 2.29 μg/mL (PLP2). FH. Fraction of *n*-hexane; FD. Fraction of dichloromethane.

## Supplementary Materials

The following are available online at https://www.mdpi.com/1424-8247/13/6/125/s1, Figure S1: Chemical structures of the proposed compounds.

## References

[1] Nadaf M., Joharchi M.R., Amiri M.S. (2019). Ethnomedicinal uses of plants for the treatment of nervous disorders at the herbal markets of Bojnord, North Khorasan Province, Iran. Avicenna J. Phytomed..

[2] Sousa S.G., Oliveira L.A., de Aguiar Magalhaes D., de Brito T.V., Batista J.A., Pereira C.M.C., de Souza Costa M., Mazulo J.C.R., de Carvalho Filgueiras M., Vasconselos D.F.P. (2018). Chemical structure and anti-inflammatory effect of polysaccharide extracted from *Morinda citrifolia* Linn (Noni). Carbohydr. Polym..

[3] Thomsen M.O., Christensen L.P., Grevsen K. (2018). Harvest strategies for optimization of the content of bioactive alkamides and caffeic acid derivatives in aerial parts and in roots of *Echinacea purpurea*. J. Agric. Food Chem..

[4] Chiou S.-Y., Sung J.-M., Huang P.-W., Lin S.-D. (2017). Antioxidant, antidiabetic, and antihypertensive properties of *Echinacea purpurea* flower extract and caffeic acid derivatives using in vitro models. J. Med. Food.

[5] Du Y., Wang Z., Wang L., Gao M., Wang L., Gan C., Yang C. (2017). Simultaneous determination of seven phenolic acids in rat plasma using UHPLC-ESI-MS/MS after oral administration of *Echinacea purpurea* extract. Molecules.

[6] Aarland R.C., Bañuelos-Hernández A.E., Fragoso-Serrano M., Del Carmen Sierra-Palacios E., Díaz de León-Sánchez F., Pérez-Flores L.J., Rivera-Cabrera F., Mendoza-Espinoza J.A. (2017). Studies on phytochemical, antioxidant, anti-inflammatory, hypoglycaemic and antiproliferative activities of *Echinacea purpurea* and *Echinacea angustifolia* extracts. Pharm. Biol..

[7] Barnes J., Anderson L.A., Gibbons S., Phillipson J.D. (2005). *Echinacea* species (*Echinacea angustifolia* (DC.) Hell., *Echinacea pallida* (Nutt.) Nutt., *Echinacea purpurea* (L.) Moench): A review of their chemistry, pharmacology and clinical properties. J. Pharm. Pharmacol..

[8] Oláh A., Szabó-Papp J., Soeberdt M., Knie U., Dähnhardt-Pfeiffer S., Abels C., Bíró T. (2017). *Echinacea purpurea*-derived alkylamides exhibit potent anti-inflammatory effects and alleviate clinical symptoms of atopic eczema. J. Dermatol. Sci..

[9] Pires C., Martins N., Carvalho A.M., Barros L., Ferreira I.C.F.R. (2016). Phytopharmacologic preparations as predictors of plant bioactivity: A particular approach to *Echinacea purpurea* (L.) Moench antioxidant properties. Nutrition.

[10] Hudson J.B. (2012). Applications of the phytomedicine *Echinacea purpurea* (Purple Coneflower) in infectious diseases. J. Biomed. Biotechnol..

[11] Senica M., Mlinsek G., Veberic R., Mikulic-Petkovsek M. (2019). Which plant part of purple coneflower (*Echinacea purpurea* (L.) Moench) should be used for tea and which for tincture?. J. Med. Food.

[12] Sultan M.T., Buttxs M.S., Qayyum M.M.N., Suleria H.A.R. (2014). Immunity: Plants as effective mediators. Crit. Rev. Food Sci. Nutr..

[13] Manayi A., Vazirian M., Saeidnia S. (2015). *Echinacea purpurea*: Pharmacology, phytochemistry and analysis methods. Pharmacogn. Rev..

[14] Khalaf A.A., Hussein S., Tohamy A.F., Marouf S., Yassa H.D., Zaki A.R., Bishayee A. (2019). Protective effect of *Echinacea purpurea* (Immulant) against cisplatin-induced immunotoxicity in rats. DARU J. Pharm. Sci..

[15] Pillai S., Pillai C., Mitscher L.A., Cooper R. (2007). Use of quantitative flow cytometry to measure ex vivo immunostimulant activity of *Echinacea*: The case for polysaccharides. J. Altern. Complement. Med..

[16] Todd D.A., Gulledge T.V., Britton E.R., Oberhofer M., Leyte-Lugo M., Moody A.N., Shymanovich T., Grubbs L.F., Juzumaite M., Graf T.N. (2015). Ethanolic *Echinacea purpurea* extracts contain a mixture of cytokine-suppressive and cytokine-inducing compounds, including some that originate from endophytic bacteria. PLoS ONE.

[17] Yang G., Li K., Liu C., Peng P., Bai M., Sun J., Li Q., Yang Z., Yang Y., Wu H. (2018). A comparison of the immunostimulatory effects of polysaccharides from tetraploid and diploid *Echinacea purpurea*. Biomed. Res. Int..

[18] Yao L., Bai L., Tan Y., Sun J., Qu Q., Shi D., Guo S., Liu C. (2019). The immunoregulatory effect of sulfated *Echinacea purpurea* polysaccharide on chicken bone marrow-derived dendritic cells. Int. J. Biol. Macromol..

[19] Sloley B.D., Urichuk L.J., Tywin C., Coutts R.T., Pang P.K.T., Shan J.J. (2001). Comparison of chemical components and antioxidant capacity of different *Echinacea* species. J. Pharm. Pharmacol..

[20] Ma Y., Kosińska-Cagnazzo A., Kerr W.L., Amarowicz R., Swanson R.B., Pegg R.B. (2014). Separation and characterization of phenolic compounds from dry-blanched peanut skins by liquid chromatography-electrospray ionization mass spectrometry. J. Chromatogr. A.

[21] Waidyanatha S., Pierfelice J., Cristy T., Mutlu E., Burback B., Rider C.V., Ryan K. (2020). A strategy for test article selection and phytochemical characterization of *Echinacea purpurea* extract for safety testing. Food Chem. Toxicol..

[22] Brown P.N., Chan M., Paley L., Betz J.M. (2011). Determination of major phenolic compounds in *Echinacea* spp. raw materials and finished products by high-performance liquid chromatography with ultraviolet detection: Single-laboratory validation matrix extension. J. AOAC Int..

[23] Vandyshev V.V., Babaeva E.Y., Drozdovskaya D.D. (2009). Triacylglycerols of the lipid fraction from fruits of two *Echinacea* species. Pharm. Chem. J..

[24] Sharma M., Vohra S., Arnason J.T., Hudson J.B. (2008). *Echinacea* extracts contain significant and selective activities against human pathogenic bacteria. Pharm. Biol..

[25] Tsai Y.-L., Chiu C.-C., Yi-Fu Chen J., Chan K.-C., Lin S.-D. (2012). Cytotoxic effects of *Echinacea purpurea* flower extracts and cichoric acid on human colon cancer cells through induction of apoptosis. J. Ethnopharmacol..

[26] Chicca A., Adinolfi B., Pellati F., Orlandini G., Benvenuti S., Nieri P. (2010). Cytotoxic activity and G1 cell cycle arrest of a Dienynone from *Echinacea pallida*. Planta Med..

[27] Graça V.C., Barros L., Calhelha R.C., Dias M.I., Carvalho A.M., Santos-Buelga C., Santos P.F., Ferreira I.C.F.R. (2016). Chemical characterization and bioactive properties of aqueous and organic extracts of *Geranium robertianum* L.. Food Funct..

[28] Graça V.C., Barros L., Calhelha R.C., Dias M.I., Ferreira I.C.F.R., Santos P.F. (2017). Bio-guided fractionation of extracts of *Geranium robertianum* L.: Relationship between phenolic profile and biological activity. Ind. Crops Prod..

[29] Bessada S.M.F., Barreira J.C.M., Barros L., Ferreira I.C.F.R., Oliveira M.B.P.P. (2016). Phenolic profile and antioxidant activity of *Coleostephus myconis* (L.) Rchb.f.: An underexploited and highly disseminated species. Ind. Crops Prod..

[30] Mascoloti Spréa R., Fernandes Â., Calhelha R.C., Pereira C., Pires T.C.S.P., Alves M.J., Canan C., Barros L., Amaral J.S., Ferreira I.C.F.R. (2020). Chemical and bioactive characterization of the aromatic plant *Levisticum officinale* W.D.J. Koch: A comprehensive study. Food Funct..

[31] Alves M.J., Ferreira I.C.F.R., Martins A., Pintado M. (2012). Antimicrobial activity of wild mushroom extracts against clinical isolates resistant to different antibiotics. J. Appl. Microbiol..

[32] Barros L., Pereira E., Calhelha R.C., Dueñas M., Carvalho A.M., Santos-Buelga C., Ferreira I.C.F.R. (2013). Bioactivity and chemical characterization in hydrophilic and lipophilic compounds of *Chenopodium ambrosioides* L.. J. Funct. Foods.

[33] Abreu R.M.V., Ferreira I.C.F.R., Calhelha R.C., Lima R.T., Vasconcelos M.H., Adega F., Chaves R., Queiroz M.-J.R.P. (2011). Anti-hepatocellular carcinoma activity using human HepG2 cells and hepatotoxicity of 6-substituted methyl 3-aminothieno[3,2-b]pyridine-2-carboxylate derivatives: In vitro evaluation, cell cycle analysis and QSAR studies. Eur. J. Med. Chem..

