# Supplementary material for: Echinacea purpurea (L.) Moench: Chemical Characterization and Bioactivity of Its Extracts and Fractions
Source: Pharmaceuticals (Basel). 2020 Jun 20;13(6):125. doi: 10.3390/ph13060125 (PMC7344755; doi:10.3390/ph13060125)
Supplement: Supplementary file 1 [file pharmaceuticals-13-00125-s001.pdf]

Figure S1. Chemical structures of the standard compounds identified in *E. purpurea*.

Ferulic acid

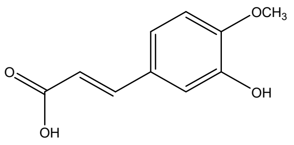

λ_{max}= 326 nm
$[M-H]^-$= 193
MS^2= 177(13),133(100),149(10)

5-*O*-caffeoylquinic acid

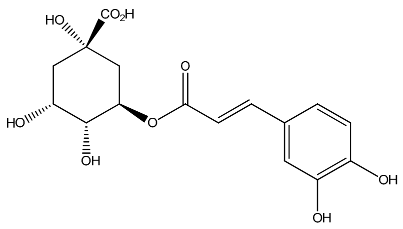

λ_{max}= 325 nm
$[M-H]^-$= 353
MS^2= 191(100),179(8),173(3),135(3)

Caffeic acid

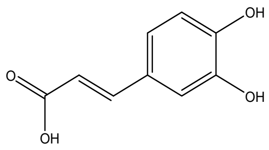

λ_{max}= 324 nm
$[M-H]^-$= 179
MS^2= 135(100)

p-Coumaric acid

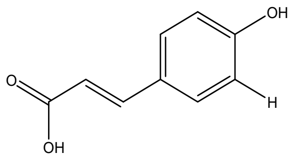

λ_{max}= 312 nm
$[M-H]^-$= 163
MS^2= 119(100)

Quercetin-3-*O*-rutinoside

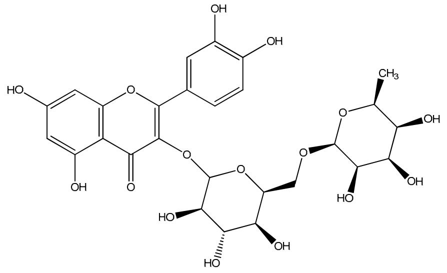

λ_{max}= 324 nm
$[M-H]^-$= 609
MS^2= 301(100)

Kaempferol-3-*O*-rutinoside

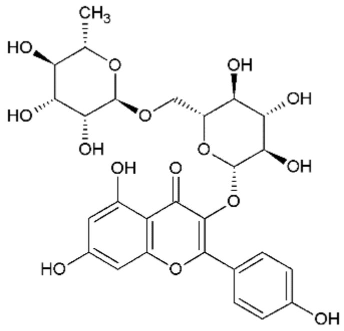

λ_{max}= 334 nm
$[M-H]^-$= 593
MS^2= 285(100)
